# Improvement in process of care and outcome in patients requiring intensive care unit admission for community acquired pneumonia

**DOI:** 10.1186/1471-2334-13-196

**Published:** 2013-04-30

**Authors:** Hugues Georges, Cécile Journaux, Patrick Devos, Serge Alfandari, Pierre Yves Delannoy, Agnès Meybeck, Arnaud Chiche, Nicolas Boussekey, Olivier Leroy

**Affiliations:** 1 Intensive Care Unit, Hôpital chatiliez, 135 rue du Président Coty, BP 619, 59208, Tourcoing, cedex, France; 2 Department of Biostatistics, Centre Hospitalier Universitaire de Lille, 59000, Lille, France; 3 Service de réanimation médicale, Hôpital chatiliez, 135 rue du Président Coty, BP 619, 59208, Tourcoing, cedex, France

**Keywords:** Severe community acquired pneumonia, Intensive care unit, Antimicrobial therapy, Combination therapy

## Abstract

**Background:**

The present study was performed to assess the prognosis of patients admitted to the intensive care unit (ICU) for community acquired pneumonia (CAP) after implementation of new processes of care.

**Methods:**

Two groups of patients with CAP were admitted to a 16-bed multidisciplinary ICU in an urban teaching hospital during two different periods: the years 1995–2000, corresponding to the historical group; and 2005–2010, corresponding to the intervention group. New therapeutic procedures were implemented during the period 2005–2010. These procedures included a sepsis management bundle derived from the Surviving Sepsis Campaign, use of a third-generation cephalosporin and levofloxacin as the initial empirical antimicrobial regimen, and noninvasive mechanical ventilation following extubation.

**Results:**

A total of 317 patients were studied: 142 (44.8%) during the historical period and 175 (55.2%) during the intervention period. Sequential Organ Failure Assessment scores were higher in patients in the intervention group (7.2 ± 3.7 vs 6.2 ± 2.8; p=0.008). Mortality changed significantly between the two studied periods, decreasing from 43.6% in the historical group to 30.9% in the intervention group (p < 0.02). A restrictive transfusion strategy, use of systematic postextubation noninvasive mechanical ventilation in patients with severe chronic respiratory or cardiac failure patients, less frequent use of dobutamine and/or epinephrine in patients with sepsis or septic shock, and delivery of a third-generation cephalosporin associated with levofloxacin as empirical antimicrobial therapy were independently associated with better outcomes.

**Conclusion:**

Positive outcomes in ICU patients with CAP have significantly increased in our ICU in recent years. Many new interventions have contributed to this improvement.

## Background

Severe community-acquired pneumonia (SCAP) is one of the leading causes of intensive care unit (ICU) admission among septic patients [1]. The mortality rate of SCAP is reportedly as high as 40% [2]. Inadequate antibiotic treatment, septic shock, acute lung injury, and associated comorbidities are the main factors contributing to this poor prognosis. In consideration of evolving epidemiologic data and the development of new antibiotics, numerous guidelines are regularly published to help physicians optimize delivery of antimicrobial treatment in patients with SCAP [3-6]. At the beginning of the previous decade, many studies reported a decrease in the mortality of ICU patients by adopting new therapeutic strategies: limitation of tidal volume and ventilatory pressure in patients with acute lung injury [7], delivery of low-dose hydrocortisone in patients with septic shock [8], use of intensive insulin therapy [9], a restrictive strategy of red cell transfusion [10], treatment with drotrecogin alpha [11] and the systematic use of noninvasive mechanical ventilation (NIMV) following extubation [12]. During the year 2004, new recommendations concerning the acute management of sepsis and septic shock were proposed in the Surviving Sepsis Campaign (SSC) guidelines [13]. Key recommendations established by international critical care and infectious disease experts included initial resuscitation, diagnostic studies, sepsis source control, antibiotic delivery, fluid administration, inotropic and vasopressor therapies, blood product administration, mechanical ventilation, steroids, and glucose control. Some of these proposals were introduced into daily practice in our unit according to this publication. During this same period, our local antibiotic policy changed to the combination of a third-generation cephalosporin (3rdGC) and antipneumococcal fluoroquinolone for the empirical treatment of SCAP. This choice was supported a few months later by the Société de Pathologie Infectieuse de Langue Française guidelines and the Infectious Diseases Society of America recommendations [5,6]. The goal of our study was to compare the outcome of patients with SCAP during 1995–2000 with that during 2005–2010, where new processes of care were implemented.

## Methods

### Setting and design

A before-and-after study design was employed. Two groups were defined. The first period included all consecutive patients with SCAP admitted between January 1995 and December 2000 (historical group). The second period included all consecutive patients with SCAP admitted between January 2005 and December 2010 (intervention group). The "4-year" hiatus corresponded to the progressive implementation of the new therapeutic strategies. The Ethics Committee of Tourcoing Hospital approved the study and waived the need for written informed consent in agreement with French regulations concerning retrospective studies (N°2011-01).

### Patients

All patients ≥ 18 years of age who presented with CAP during the two studied periods were included. CAP was defined as the presence of a new pulmonary infiltrate on a chest radiograph at the initial presentation or that occurring within 48 hours following hospitalization along with the acute onset of two or more of the following signs or symptoms: fever, new or increasing cough or sputum production, dyspnea, pleuritic chest pain, new focal signs on chest examination, and leukocytosis or leukopenia as defined by local laboratory values. Patients were admitted to the ICU for mechanical ventilation (MV), for vasoactive drug support, or because the clinician believed that the individual was otherwise unstable and required intensive medical care. Patients were excluded for the following reasons: diagnosis of aspiration pneumonia, diagnosis of pneumocystis pneumonia, and if the Pneumonia Severity Index (PSI) score were ≤III [14]. For patients with more than one admission for CAP in our unit, only the first admission was taken into account.

### Data collection

Collected baseline characteristics were age, gender, alcoholism, chronic obstructive pulmonary disease (COPD), congestive heart failure, cancer, immunosuppression, functional status, and diabetes mellitus. The diagnosis of COPD was based on the clinician’s assessment and included data provided by patients or relatives and found on previous medical reports. Congestive heart failure was defined as New York Heart Association (NYHA) class IV [15]. Functional status was assessed by the Knaus score [16]. Immunosuppression was defined as a recent use of immunosuppressants or systemic corticosteroids (i.e., prednisolone at >0.5 mg/kg/day for more than 1 month), human immunodeficiency virus infection, neutropenia (absolute neutrophil count of <1000 cells/mm^3^), organ transplantation with ongoing immunosuppressants, or cancer chemotherapy within the past 3 months. Patients with cancer were defined as those presenting with a diagnosis of a solid tumor or hematologic malignancy within 5 years of ICU admission.

Clinical and biological parameters collected upon ICU admission were the Simplified Acute Physiology Score (SAPS II) [17], Sequential Organ Failure Assessment (SOFA) score [18], PSI risk class, admission to the ICU within 24 hours after hospital admission, leukopenia (leukocyte count of <4000/mm^3^), PaO2:FIO2 in patients undergoing MV, an etiological diagnosis of pneumonia, and associated bacteremia. An etiologic diagnosis was considered when pathogens were isolated from (1) blood cultures, (2) pleural fluid, (3) sputum samples (bacterial growth in culture of endotracheal aspiration sample of ≥10^5^cfu/mL in intubated patients), (4) a four-fold rise in IgG titers for *Legionella pneumophila*, *Chlamydia pneumoniae,* or *Mycoplasma pneumoniae*, (5) a single increased IgM titer for *M. pneumoniae* (≥1:16), *Chl. pneumoniae* (≥1:32), or *Coxiella burnetii* (≥1:64) or (6) positive urinary antigens for *L. pneumophila* type 1 or *Streptococcus pneumoniae* (tests performed during the second period of the study).

The recorded data on therapeutics performed within 48 hours after ICU admission were the class of delivered antimicrobial agents, antimicrobial combination therapy, adequacy of antimicrobial therapy, institution of antimicrobial therapy within 8 hours after hospital admission, NIMV and/or IMV, crystalloid or colloid fluid administration (first 24 hours after ICU admission), and requirement and nature of vasoactive agents. Antimicrobial therapy was considered to be adequate if the causative pathogens were susceptible to at least one of the delivered antimicrobial agents as shown on the antibiotic susceptibility reports. The recorded vasoactive drugs were dobutamine, dopamine, norepinephrine and epinephrine.

The recorded data on characteristics of the ICU stay were the mean tidal volume during the first 3 days of MV, transfusion requirement, occurrence of nosocomial infection, duration of MV, duration of ICU stay, and ICU mortality. Recorded nosocomial infections were ventilator-associated tracheobronchitis, ventilator-associated pneumonia, and bacteremia. Ventilator-associated tracheobronchitis, ventilator-associated pneumonia were defined according to previous reports [19,20].

### Standard process of care during the two studied periods

#### Historical period

Vasoactive drugs: Pulmonary arterial catheterization was performed in patients with septic shock. The choice of vasopressor agents was left to each physician. Dobutamine was prescribed to increase oxygen delivery because it was the practice at that time [21].

Fluid therapy: Intravascular volume loading was assessed by physical examination and/or pulmonary arterial catheterization.

Insulin therapy: Insulin therapy was only prescribed for patients with diabetes mellitus who required insulin therapy at home.

MV: A tidal volume of 8 to 10 mL/kg was delivered after starting MV. The tidal volume was then modified according to the acid- base status, without specific limitations on the increase in the tidal volume.

Transfusion requirement: A hemoglobin level of ≥10 g/dL was required because of its widespread use in clinical medicine [22].

NIMV following extubation: NIMV was only used as rescue therapy when respiratory distress followed extubation.

Antimicrobial therapy: Our local hospital policy suggested treatment of SCAP with a beta-lactam plus fluoroquinolone according to the French recommendations published at the time [23]. The beta-lactam was chosen by physicians among amoxicillin (50 mg/kg/d), amoxicillin/clavulanate (50 mg/kg/d), cefotaxime (50 mg/kg/d), or ceftriaxone (2 g/d). The fluoroquinolone was similarly chosen between ofloxacin (400 mg/d) or ciprofloxacin (800 mg/d).

#### Intervention period

New processes of care were progressively introduced in the unit during the period 2001^_^2004. An educational program based on the SSC guidelines was next implemented in our unit over a 1-month period (December 2004). The procedures were then written and made available to each physician in the unit.

Vasoactive drugs: The recommended vasopressor was norepinephrine. Dobutamine was only considered for patients with a low cardiac output in the presence of adequate left ventricular filling pressure and adequate mean arterial pressure as schown by echocardiography. The use of dobutamine for supranormal oxygen delivery was discontinued.

Fluid therapy: Intravascular volume loading was assessed by physical examination and/or arterial pulse pressure and/or echocardiography. Fluid responsiveness was determined by respiratory variations in the superior and inferior vena cava diameters, left ventricular stroke, and arterial pulse pressure [24].

Stress-dose steroid: Intravenous hydrocortisone was instituted in all septic patients who required vasopressor use.

Transfusion requirement: A restrictive red blood cell transfusion strategy was adopted targeting a hemoglobin level of 7 to 9 g/dL in all patients with the exception of those with acute myocardial infarcts or unstable angina, for whom a level of 10 g/dL level was required.

Low tidal volume: A tidal volume of 6 mL/kg was started in all patients undergoing MV. The tidal volume was then modified following blood gas analysis. Maintenance of an end-inspiratory plateau pressure of no more than 30 cm of H2O was a strict goal.

Insulin therapy: Insulin therapy was started in all patients to maintain a blood glucose level of <150 mg/dL.

NIMV: After a successful spontaneous breathing trial, NIMV was instituted within the next 24 hours following extubation procedures in patients with severe COPD (long-term oxygen therapy and noninvasive ventilation at home) and in patients with chronic cardiac failure (NYHA class IV).

Antimicrobial therapy: Between the two studied periods, our hospital guideline for empirical antimicrobial therapy in patients with CAP was modified according to international recommendations [25]. A combination of a 3rdGC (cefotaxime or ceftriaxone) and a respiratory fluoroquinolone (levofloxacin) was suggested. Levofloxacin was discontinued after 3 days of delivery if *Legionella* urinary antigen tests remained negative.

#### Years 2001–2004

Age, SAPS II, number of patients with MV or vasopressor support, and mortality for all CAP patients with CAP admitted to our unit during this period were reported.

### Statistical analysis

The two studied groups were compared using the chi-squared test or Fisher's exact test for categorical parameters and Student’s *t* test for continuous variables. When appropriate, continuous variables were analyzed as categorical variables using clinically meaningful cut-off points.

The first objective of the study was to assess the mortality of patients during the two studied periods.

The second objective was to determine prognostic factors associated with outcome. We performed a univariate analysis including all patients to assess risk factors for mortality. Differences between the two studied groups were considered to be significant for variables yielding a p value of ≤0.05. To study the relationship between mortality and any predictor while taking the significant variables into account, we performed three stepwise logistic regressions as follows. The significant variables collected within 48 hours of admission were used for the first analysis, the therapeutic procedure instituted upon ICU admission and during the ICU stay were used for the second analysis, and all significant variables were entered into a third stepwise analysis. A level of p ≤0.15 was chosen for covariate retention.

The third objective of the study was to analyze the evolution of the SOFA score during the first week of ICU admission with a linear mixed model.

All statistical analyses were performed using the SAS Software, V9.1.

## Results

### Patients

A total of 317 patients were admitted to our ICU for CAP during the two studied periods: 142 (44.8%) during the historical period and 175 (55.2%) during the intervention period. Baseline characteristics and presentation upon ICU admission in the two groups are reported in Table 1.

**Table 1:** Management of patients with severe CAP

	**Historical**	**Intervention**	
	**Group**	**Group**	***p***
	**n=142 (44.8)**	**n=175 (55.2)**	
Patients characteristics			
Age, yrs	65.7 ± 14.4	64.2 ± 15.7	0.39
Male, n (%)	109 (75.7)	114 (65.1)	0.02
Alcoholism, n (%)	39 (27.4)	48 (27.4)	0.99
COPD, n (%)	69 (48.6)	63 (36)	0.02
Chronic cardiac failure, n (%)	19 (13.4)	27 (15.4)	0.60
Diabetes mellitus, n (%)	15 (10.6)	28 (16)	0.15
Cancer, n (%)	4 (2.8)	14 (8)	0.04
Immunosuppression, n (%)	8 (5.6)	34 (19.4)	0.0003
Knaus score, n (%)			0.18
A	11 (7.7)	21 (12)	
B	45 (31.7)	69 (39.4)	
C	74 (52.1)	72 (41.4)	
D	12 (8.4)	13 (7.3)	
ICU admission			
Within 24 hrs of hospital admission, n (%)	112 (78.9)	127 (72.6)	0.19
SAPS II	47.8 ± 16.3	48.7 ± 19.7	0.67
SOFA score	6.2 ± 2.8	7.2 ± 3.7	0.008
PSI risk class V, n (%)	93 (65.5)	118 (67.4)	0.71
Leukopenia, n (%)	17 (12)	13 (7.4)	0.16
Hemoglobin level (g/dL)	12.7 ± 2.5	11.8 ± 2.3	0.001
PaO2:FIO2 < 200 in patients with MV, n (%)	84 (78.5)	85 (66.4)	0.04
Documented pneumonia, n (%)	86 (60.6)	100 (57.1)	0.53
Pathogens*			
*Streptococcus pneumoniae, n (%)*	45 (52.3)	63 (63)	0.42
*Streptococcus pneumoniae with DSP, n (%)*	18 (20.9)	17 (17)	0.40
*Staphylococcus aureus, n (%)*	12 (13.9)	5 (5)	0.02
*Enterobacteriacea, n (%)*	11 ( 12.8)	12 (12)	0.76
*Haemophilus Influenzae, n (%)*	12 (13.9)	11 (11)	0.45
*Pseudomonas aeruginosa, n (%)*	4 (4.6)	2 (2)	0.29
*Legionella pneumophila, n (%)*	1 (1.1)	7 (7)	0.06
Other, n (%)	6 (6.9)	7 (7)	0.91
Bacteremia, n (%)	19 (13.6)	34 (19.4)	0.16

Results are mean ± SD unless indicated otherwise.

COPD, chronic obstructive pulmonary disease; ICU, intensive care unit.

SAPS, simplified acute physiology score; SOFA, sequential organ failure assessment score.

PSI, pneumonia severity index; DSP, decreased susceptibility to penicillin.

* Some pneumonia are caused by many pathogens.

Comparison upon ICU admission between historical controls (1995^_^2000) and current strategies (2005^_^2010).

A total of 127 patients with CAP were admitted to our ICU during the years 2001–2004.

Mean age and mean SAPS II were 65.1 ± 15.3 years and 50.5 ± 19.7, respectively. Among these patients, 93 (73.2%) underwent MV and 66 (51.9%) required vasopressor support. Fifty-three (41.7%) patients died.

### Process of care

Therapeutic interventions upon ICU admission and during the ICU stay are reported in Table 2. As expected, the intervention period was characterized by MV with a lower tidal volume, lower transfusion requirement, more intravascular volume expansion, lower use of dobutamine, more frequent postextubation NIMV procedures and more frequent norepinephrine use in patients with septic shock. Low-dose steroid administration was instituted in 81.6% of patients requiring vasoactive drugs, and intensive insulin therapy was delivered in 86.3% of patients.

**Table 2:** Management of patients with severe CAP

	**Historical**	**Intervention**	
	**Group**	**Group**	***p***
	**n=142 (44.8)**	**n=175 (55.2)**	
NIMV within 48 hrs of admission, n (%)	26 (18.3)	35 (20)	0.7
MV within 48 hrs of admission, n (%)	106 (74.6)	127 (72.6)	0.18
Fluid administration within 24 hours of admission, ml	1300 ± 1900	2200 ± 1700	< 0.0001
Vasoactive drugs within 48 hrs of admission, n (%)	74 (52.2)	109 (62.3)	0.07
Dobutamine, n (%)	46 (32.4)	17 (9.7)	< 0.0001
Dopamine, n (%)	8 (5.6)	10 (5.7)	0.97
Norepinephrine, n (%)	19 (13.4)	105 (57.7)	< 0.0001
Epinephrine, n (%)	20 (14.1)	11 (6.3)	0.02
Dual therapy, n (%)	121 (85.2)	158 (90.3)	0.16
Adequate antimicrobial therapy/ documented pneumonia, n (%)	82 (96.4)	92 (92)	0.20
Antimicrobial therapy within 8 h of admission, n (%)	109 ( 76.7)	138 (78.8)	0.65
Combination of a 3rdGC and levofloxacin, n (%)	0	115 (65.7)	< 0.0001
Insulin therapy, n (%)	19 (13.4)	151 (86.3)	
Intensive insulin therapy, n (%)	0	151 (86.3)	< 0.0001
Mean tidal volume, mL/kg	8.17 ± 1.19	6.64 ± 0.73	< 0.0001
Low-dose steroid administration, n (%)	0	89 (50.8)	< 0.0001
Transfusion, n (%)	62 (43.7)	40 (22.8)	< 0.0001
Number of red blood cell units transfused per patient	2.6 ± 4.5	0.8 ± 2.1	< 0.0001
Systematic postextubation NIMV, n (%)	8 (5.6)	20 (11.4)	0.07

Results are mean ± SD unless indicated otherwise.

NIMV, non invasive mechanical ventilation; MV, mechanical ventilation.

3rdGC, third generation cephalosporin.

Therapeutics interventions during the two studied periods, historical controls (1995–2000) and current strategies (2005–2010).

Antibiotic administration in each period is presented in Table 3. Among beta-lactams, 3rdGCs were more frequently used than aminopenicillins during the intervention period (78% vs. 50.4%, p < 0.001).

**Table 3:** Management of patients with severe CAP

	**Historical**	**Intervention**
	**Group**	**Group**
	**n=142 (44.8)**	**n=175 (55.2)**
Monotherapy	21	17
Amoxicillin, n=	4	2
Amoxicillin clavulanate, n=	10	5
Cetotaxime or Ceftriaxone, n=	7	7
Other, n=	-	3
Combination therapy	121	158
Amoxicillinc and Ofloxacin, n=	10	0
Amoxicillin and Ciprofloxacin, n=	1	0
Amoxicillin clavulanate and Ofloxacin, n=	29	4
Amoxicillin clavulanate and Ciprofloxacin, n=	11	0
Cetotaxime or Ceftriaxone and Ofloxacin, n=	49	9
Cetotaxime or Ceftriaxone and Ciprofloxacin, n=	5	0
Amoxicillin and Levofloxacin, n=	0	1
Amoxicillin clavulanate and Levofloxacin, n=	0	12
Cetotaxime or Ceftriaxone and Levofloxacin, n=	0	115
Other combination, n=	16	17

Antibiotics delivered according to studied period; historical controls (1995–2000) and current strategies (2005–2010).

### Outcome and prognostic factors

**Figure 1:**
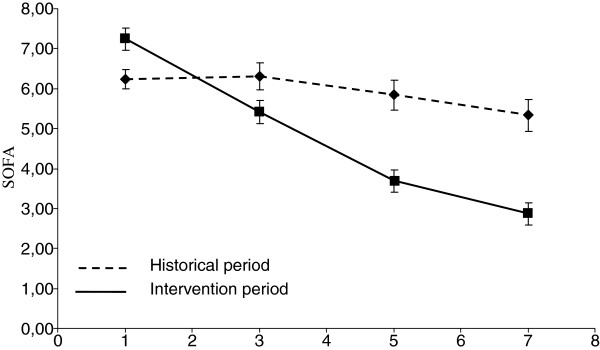
Evolution of the SOFA score during the first week following ICU admission.

Between the two studied populations, 116 (36.6%) patients died. The prognosis of patients improved during the intervention period: 62 (43.6%) patients died during the historical period, and 54 (30.9%) patients diedduring the intervention period (p < 0.02). Mortality remained high during the years 2001–2004; 53 (41.7%) patients with CAP died. Significant prognostic factors between survivors and nonsurvivors are reported in Table 4. A reduction in the evolution of the SOFA score during the first week following ICU admission was noted during the intervention period (p < 0.001) (Figure 1). The duration of MV, duration of ICU stay and occurrence of nosocomial infection were not different between the historical and intervention periods (10.7 ± 13.3 vs.10.8 ± 13.7 days, p = 0.96; 13.4 ± 14.4 vs 13.2 ± 13.6 days, p = 0.88; and 41 (20.4%) vs. 32 (27.6%), p = 0.14, respectively).

**Table 4:** Significant prognostic factors derived from univariate analysis

	**Survivors**	**Non survivors**	
	**n = 201**	**n = 116**	***p***
Patient characteristics			
Age, yrs	62.9 ± 15.3	68.5 ±14.4	0.001
ICU admission			
SAPS II	40.8 ± 12.0	61.3 ± 19.9	< 0.001
SOFA score	5.7 ± 5.3	8.6 ± 3.5	< 0.001
PSI class V, n (%)	108 (53.7)	103 (88.8)	< 0.001
Leukopenia, n (%)	10 (5)	20 (17.2)	< 0.001
MV within 48 hrs of admission, n (%)	124 (61.7)	109 (94)	< 0.001
PaO2:FIO2 < 200 in patients with MV, n (%)	76 (37.8)	93 (80.2)	< 0.001
Therapeutics interventions			
Vasoactive drugs within 48 hrs of admission, n (%)	88 (43.8)	95 (81.9)	< 0.001
Dobutamine, n (%)	26 (12.9)	37 (31.9)	< 0.001
Dopamine, n (%)	7 (3.5)	11 (9.5)	0.02
Norepinephrine, n (%)	66 (32.8)	54 (46.5)	0.01
Epinephrine, n (%)	7 (3.5)	24 (20.7)	< 0.001
Combination of a 3rdGC and levofloxacin, n (%)	84 (41.8)	31 (26.7)	0.007
Mean tidal volume, mLl/kg	7.14 ± 1	7.56 ± 1.41	0.008
Low-dose steroid administration, n (%)	47 (23.3)	42 (36.2)	0.01
Transfusion, n (%)	48 (23.9)	54 (46.5)	< 0.001
Systematicpost extubation NIMV, n (%)	21 (10.4)	7 (6)	0.01
Intervention period, n (%)	121 (69.1)	54 (30.8)	0.01

Results are mean ± SD unless indicated otherwise.

### Multivariate analysis

Results are presented in Table 5. Admission during the intervention period was independently associated with a better prognosis (p = 0.001). The multivariate analyses underscore several poor prognostic factors related to pneumonia severity upon ICU admission: SAPS II > 50, requirement for vasoactive drugs, and a PSI risk class of V. Among therapeutic procedures, dobutamine and adrenaline use, low-dose steroid administration, and a transfusion requirement were associated with a worse prognosis. In contrast, administration of the combination of a 3rdGC and levofloxacin as an empirical antimicrobial regimen and systematic postextubation NIMV improved outcomes.

**Table 5:** Management of patients with severe CAP

**Variables**	**Adjusted OR**	**95% CI**	**p Value**
**Admission data**			
SAPS II > 50	4.21	2.35-7.56	< 0.0001
Vasoactive drugs within 48 hrs of admission	2.91	1.51-5.61	0.0001
PSI class V	2.70	1.3- 5.6	0.007
Historical period	2.53	1.42-4.52	0.001
**Therapeutics interventions**			
Epinephrine use	4.97	1.96-12.6	0.0007
Low-dose steroid administration	2.29	1.18-4.46	0.01
Dobutamine	2.12	1.09-4.11	0.02
Transfusion	1.92	1.07-3.44	0.02
Combination of a 3rdGC and levofloxacin	0.37	0.19-0.74	0.004
Systematic postextubation NIMV	0.30	0.11-0.82	0.01
**Whole data**			
SAPS II > 50	3.51	1.89-6.51	< 0.0001
PSI risk class V	2.73	1.23-6.04	0.01
Vasoactive drugs within 48 hrs of admission	2.48	1.21-5.08	0.01
Transfusion	1.97	1.06-3.65	0.03
Combination of a 3rdGC and levofloxacin	0.36	0.18-6.51	0.002
Systematic postextubation NIMV	0.34	0.12-0.95	0.03

Multiple logistic regressions of factors associated with poor outcome.

## Discussion

The main result of our study is that in our unit, the mortality of patients with CAP was reduced from 43.6% to 30.9%. We demonstrated that compliance with some components of the bundles derived from the SSC guidelines was associated with a significant improvement in the outcome of patients with CAP. Our report confirms the feasibility of changing the quality of care in patients with severe sepsis or septic shock using new, easily applicable therapies. In the same way, our study supports the hypothesis that the use of empirical antimicrobial therapy concordant with national clinical practices is associated with improvement in outcomes.

The mortality in the first period may appear high compared with that in some studies published at the same time [26,27]. However, our patients were more severely ill; most required MV and exhibited septic shock. Two recent studies evaluating the clinical and economic burden of CAP in Europe reported an ICU mortality reaching 45% [2,28]. Our results are even more significant considering that the severity of disease upon ICU admission was nearly identical between the two groups. There were no differences in SAPS II, PSI class V, leukopenia, bacteremia, delay of ICU admission, use of MV, or vasoactive drug support. SOFA scores were significantly higher in the intervention group. Causative pathogens changed little during the two periods. The incidence of penicillin-nonsusceptible *S. pneumoniae* did not vary. *Staphylococcus aureus* infection was more frequent in the historic group but probably had no impact on outcome because no strains were methicillin-resistant.

The combination of a 3rdGC with a respiratory fluoroquinolone is among the new interventions associated with improvement in outcomes. This association seems attractive, allowing for the delivery of two antipneumococcal drugs with high bactericidal activity and excellent tissue penetration. Drago recently reported that among numerous combinations of fluoroquinolones or macrolides, the combination of levofloxacin with a parenteral cephalosporin was the most active against *S. pneumoniae* strains [29]. The antipneumococcal activity of levofloxacin could explain the improvement in outcomes; the patients benefited from a “true” combination therapy against the main causative pathogen of CAP. This association could be a major advantage in the most severe forms of pneumococcal disease. This result confirms that of a previous report in which we showed that when combined with beta-lactams, levofloxacin is associated with lower mortality than ofloxacin or ciprofloxacin in patients with severe pneumococcal CAP [30]. Other studies have reported opposing results with a combination of beta-lactams and fluoroquinolones, but studied patients, delivered fluoroquinolones (ciprofloxacin) and treatment duration of fluoroquinolones were not identical [31,32]. The first-line empirical beta-lactam differed between the two studied periods in that a 3rdGC was used in place of aminopenicillins. In both cases, however, the drug used was the drug recommended for the treatment of CAP at the time [3,23]. Our results support the most recent Infectious Diseases Society of America guideline suggesting that the combination of levofloxacin and a beta-lactam represents a better option with which to treat patients with CAP in the ICU (level I evidence) [5].

The use of NIMV as a systematic extubation and weaning technique was first proposed in patients with chronic respiratory failure [12]. Subsequent studies proposed the use of NIMV in patients with risk factors for respiratory failure after extubation [33,34]. NIMV offsets muscle fatigue and tachypnea, increases tidal volume and reduces intrinsic positive and expiratory pressure. During the intervention period of the present study, we applied NIMV after a successful T-piece weaning trial in extubated patients with severe underlying COPD or severe chronic cardiac failure. When this procedure was well tolerated, NIMV was delivered by face mask, spaced by periods of spontaneous ventilation, for at least 24 hours.

Many studies have indicated that blood transfusion in critically ill patients is associated with worse outcome [35]. Critically ill patients may be at increased risk for the immunosuppressive and microcirculatory complications of blood transfusion. In the absence of acute bleeding, hemoglobin levels of 70 to 90 g/L are well tolerated by most critically ill patients and a transfusion threshold of 70 g/L is now considered appropriate. In our study, despite a higher average hemoglobin concentration in the historical group, the number of patients transfused and the number of red blood cell units transfused per patient were higher in this group. Our results support the potential benefits of a restrictive transfusion strategy in patients with severe sepsis [10].

During the intervention period, 81.6% of patients with septic shock received steroids. Low-dose steroid administration is associated with a worse prognosis. The use of corticosteroids in the treatment of sepsis or septic shock remains controversial [8,36]. Discontinuation of steroids could lead to a rebound of the inflammatory response and an increased incidence of rebound sepsis. We did not perform a 250-μg ACTH stimulation test to identify responders; thus, steroids were probably overused, notably in patients who received low-dose vasopressor therapy. In 2008, the second SSC guidelines recommended the use of steroid therapy only when blood pressure is poorly responsive to fluid replacement and vasopressor therapy [37].

Norepinephrine administration was more frequent and dobutamine administration was less frequent during the intervention period than during the historical period. In 2004, Leone reported a lack of standardization in the use of catecholamines by French physicians [38]. Our study shows an association between the use of epinephrine and unfavorable outcomes. Human and animal studies have suggested that epinephrine has deleterious effects on the splanchnic circulation [39].

Our study has limitations. First, our analysis was retrospective, and the possibility that the type of patients selected changed over time cannot be excluded. Second, the high number of changes in practice throughout the time course of the study limits our ability to determine a causal relationship between new therapeutic interventions and the outcome we evaluated. Most notably, some therapeutics, such as the combination of a 3rdGC and levofloxacin have been prescribed only recently, and studies with a different methodological approach are necessary to confirm our results. Third, pneumonia severity upon ICU admission was higher in the second period, and we cannot exclude the possibility that the change in ICU triage and admission practice patterns between the two time periods caused patients at higher risk of imminent death to be triaged away from ICU care at a higher rate. However, this probably concerned few patients. Indeed, life-sustaining treatments and palliative care for critically ill patients are generally delivered in our unit rather than in the emergency department. Fourth, the medical staff experienced substantial turnover during the studied periods and we cannot exclude the possibility that staff members were more experienced during the intervention stage. Fifth, this was a single-center study, and these results may not be extrapolated to other hospitals. Finally, our study predominantly applies to elderly male patients, this demographic was the most commonly represented.

Because mortality remains high, other therapies as well as a more rapid diagnosis and management in the emergency department are necessary. The anti-inflammatory effects of statins, the use of biomarkers to predict early treatment failure, and high-flow nasal therapy to reduce intubation require further study.

## Conclusions

Management of patients with CAP requiring ICU admission has evolved in recent years. In our unit, many new procedures concerning associated sepsis management and delivery of empirical antimicrobial therapy have been instituted according to successive published guidelines. These implementations were accompanied by improvements in outcomes. Delivery of a the combination of a 3rdGC and levofloxacin as initial empirical antimicrobial therapy and a restrictive transfusion strategy targeting a hemoglobin level of 7 to 9 g/dL are the main new therapeutic procedures contributing to this improvement.

## Competing interest

The authors declare that they have no competing interest.

## Authors’ contributions

HG and CJ conceived the study and wrote the paper. PD performed the statistical analysis. SA revised the manuscript. PYD, AM, AC, NB and OL prepared and collected data. All authors read and approved the final manuscript.

## Pre-publication history

The pre-publication history for this paper can be accessed here:

http://www.biomedcentral.com/1471-2334/13/196/prepub
